# When viral infections meet the anti-MDA5 antibody-positive dermatomyositis

**DOI:** 10.3389/fimmu.2025.1649489

**Published:** 2026-01-07

**Authors:** Shuzi Liu, Zheng Zhao, Yabin Li, Yao Tan, Huabin Tian, Fei Xie

**Affiliations:** 1 Department of Respiratory and Critical Care Medicine, The First Medical Center, Chinese People’s Liberation Army (PLA) General Hospital, Beijing, China; 2 Department of Rheumatology and Immunology, The First Medical Center, Chinese People’s Liberation Army (PLA) General Hospital, Beijing, China; 3 State Key Laboratory of Biomacromolecules, Institute of Biophysics, Chinese Academy of Sciences, Beijing, China

**Keywords:** anti-MDA5 antibody, dermatomyositis, interstitial lung disease, pathogenesis, SARS-CoV-2, viral infections

## Abstract

Anti-melanoma differentiation-related gene 5 (MDA5) antibody-positive dermatomyositis (anti-MDA5^+^ DM) is recognized as a distinct subtype of dermatomyositis, characterized by its frequent association with interstitial lung disease (ILD), particularly rapidly progressive ILD (RP-ILD), which is associated with a poor prognosis and high mortality. MDA5 functions as a cytoplasmic sensor for viral double-stranded RNA. The expression level of anti-MDA5 antibodies is positively correlated with disease severity. Notably, anti-MDA5 antibodies have been detected in patients infected with SARS-CoV-2. While the mechanisms underlying the generation of anti-MDA5 antibodies and their pathogenic role remain incompletely understood, accumulating data support the hypothesis that viral infections may trigger the production of these antibodies. This review provides a comprehensive analysis of the interplay between anti-MDA5 antibodies and viral infections in patients with anti-MDA5^+^ dermatomyositis (DM), with a focus on the potential mechanisms by which viral infections induce autoantibody formation.

## 1 Introduction

Dermatomyositis (DM) is classified into five distinct subtypes based on muscle-specific autoantibodies (MSAs), including nuclear matrix protein (NXP) 2, transcription intermediary factor (TIF)1-γ, nucleosome-remodeling and deacetylase complex (also known as Mi2), melanoma differentiation-associated gene 5 (MDA5), and small ubiquitin-like modifier-activating enzyme (SAE) (1). The anti-MDA5 antibody was first identified by Sato et al. in 2005 (2). Patients with anti-MDA5-positive DM (anti-MDA5^+^ DM) are characterized by four primary clinical features: the presence of anti-MDA5 antibodies, skin ulcers, muscle weakness or absence of myositis, and interstitial lung disease (ILD), particularly rapidly progressive ILD (RP-ILD) (3, 4). During the first six months following diagnosis, especially within the initial three-month period, these patients demonstrate markedly increased risks of developing RP-ILD and mortality, frequently necessitating admission to the respiratory intensive care unit (ICU) (5). The substantial morbidity and mortality related to RP-ILD pose a significant threat to patient survival.

A critical question that warrants further investigation is why anti-MDA5^+^ DM patients are more susceptible to life-threatening RP-ILD than patients with antisynthetase syndrome (ASS) or other DM subtypes. MDA5, which functions as an RNA sensor, serves as a critical pattern recognition receptor for severe acute respiratory syndrome coronavirus 2 (SARS-CoV-2) (6). The extent to which SARS-CoV-2, herpesviruses, or other viral pathogens contribute to the initiation and progression of ILD or RP-ILD remains unclear. Previous studies have established correlations between anti-MDA5 antibody titers and disease severity as well as prognosis (7–9). However, the mechanisms governing anti-MDA5 antibody generation, the causal link between viral infection and antibody production, and the pathogenic roles of these antibodies in anti-MDA5^+^ DM require further elucidation.

Importantly, despite intensive glucocorticoid and immunosuppressant therapy, patients with anti-MDA5^+^ DM-related ILD/RP-ILD generally demonstrate poor treatment responses and dismal prognoses (3), posing a substantial challenge to clinicians. This clinical entity has attracted significant attention within the medical community. Nevertheless, the exact etiology and pathogenesis of anti-MDA5^+^ DM-related ILD or RP-ILD remain undefined. This review seeks to provide a comprehensive synthesis of two pivotal factors in anti-MDA5^+^ DM: anti-MDA5 antibodies and viral infections.

## 2 Anti-MDA5 antibody-positive dermatomyositis: viral infection

### 2.1 Earliest theory: anti-MDA5^+^ DM shows seasonal distribution characteristics

Epidemiological investigations conducted in Japan, China, and France have revealed distinct seasonal patterns in disease onset among individuals with anti-MDA5^+^ DM. Notably, incidence rates peak during the winter and spring months (October to March), whereas they reach a nadir during the summer months (July to September) (10–13). This seasonal trend aligns with periods of heightened respiratory viral infection activity, suggesting that viral infections may serve as a trigger for anti-MDA5^+^ DM.

In 2011, the first epidemiological study on anti-MDA5^+^ DM was conducted by Muro et al. in Japan. Their results demonstrated that anti-MDA5^+^ DM patients were predominantly clustered in rural areas adjacent to river systems, with significantly lower case frequencies observed in urban environments. Coxsackievirus B, a small RNA virus, was detected in these aquatic ecosystems. Subsequent analysis revealed that in regions with lower population density (fewer than 108,000 residents), the prevalence of anti-MDA5 antibodies peaked in autumn; conversely, no comparable seasonal trends were identified in highly populated areas (exceeding 130,000 residents) (10). Hosono et al. subsequently reported the identification of a novel autoantibody against the SFPQ protein in anti-MDA5^+^ DM patients. The SFPQ protein was recognized for its role in the innate immune response. The study revealed that both patient diagnoses and the appearance of anti-SFPQ antibodies exhibited seasonal patterns, suggesting that environmental factors may influence the production of autoantibodies. Notably, in a cohort of 27 patients who were double-positive for anti-MDA5 and anti-SFPQ antibodies, no disease onset occurred during the summer months (June to July) (14).

A 2020 large-scale multicenter study in Japan by Nishina et al. demonstrated that anti-MDA5^+^ DM-related ILD predominantly occurred between October and March of the following year, with the majority of patients residing near freshwater habitats. In contrast, no significant seasonal variations were observed in individuals with anti-aminoacyl-tRNA synthetase (anti-ARS) antibodies or those negative for both antibody types (11). Toquet et al.’s 2021 study in France revealed a significant increase in the incidence of anti-MDA5^+^ DM among French patients during the springtime (12). A 2022 multicenter retrospective cohort study in Hong Kong, China, by Ho et al., reported a marked increase in anti-MDA5^+^ DM-related RP-ILD from October to December, with further analysis implicating infections as a contributing factor to the development of RP-ILD (13). Given the established association between infectious respiratory diseases and the risk of idiopathic inflammatory myopathy (IIM) (15), these findings suggest that environmental factors, particularly seasonally and geographically specific viral infections, may play a substantial role in the pathogenesis of anti-MDA5^+^ DM.

### 2.2 Anti-MDA5^+^ DM and SARS-CoV-2 infection

#### 2.2.1 Presence of anti-MDA5 antibodies in SARS-CoV-2 infected individuals

Extensive research has been conducted on coronavirus disease 2019 (COVID-19), an illness caused by the highly transmissible SARS-CoV-2. This seasonal pattern aligns with periods of elevated respiratory viral infection activity, suggesting that viral infections may act as a trigger for anti-MDA5^+^ DM. Studies have investigated the positivity rates and titers of anti-MDA5 antibodies in association with clinical manifestations and disease progression. Additionally, early detection of anti-MDA5 antibodies could serve as a biomarker for identifying high-risk patients and predicting disease outcomes (16). However, in the retrospective cohort study by Wang et al. (16), while anti-MDA5 antibody titers showed statistically significant elevation among severe/non-surviving patients, the absolute differences between groups were modest, with substantial overlap in distributions. This suggests that anti-MDA5 antibodies may act as a nonspecific marker of systemic immune activation rather than a direct driver of disease severity of COVID-19. Future studies should integrate anti-MDA5 antibody titers with multi-omics data and clinical phenotypes to better define their role in risk stratification.

Quintana-Ortega et al. (17) reported the first documented case of a fatal outcome in a juvenile dermatomyositis (JDM) patient with severe RP-ILD complicated by SARS-CoV-2 infection (17). In a large-scale observational study conducted in Yorkshire, UK, David et al. (6) analyzed 15 MSAs and revealed that the increase in anti-MDA5 autoantibody detection corresponded with the peak of SARS-CoV-2 transmission (6, 18). However, the exact mechanisms underlying the generation of anti-MDA5 antibodies in individuals infected with SARS-CoV-2 remain undefined, as does the direct role of these antibodies in the pathogenesis of COVID-19.

#### 2.2.2 Overlap between Anti-MDA5^+^ DM and SARS-CoV-2-infected individuals

There is substantial overlap in the clinical characteristics and pathogenic mechanisms of patients with anti-MDA5^+^ DM and SARS-CoV-2 infections. Comprehensive investigations into the pathophysiological processes of SARS-CoV-2-infected individuals may provide critical insights that deepen our understanding of the pathogenesis of anti-MDA5^+^ DM.

Both anti-MDA5^+^ DM patients and SARS-CoV-2-infected individuals are predisposed to developing concurrent ILD, particularly RP-ILD, which can lead to acute respiratory distress syndrome (ARDS) and mortality (19). Interstitial pneumonia induced by both conditions results in nearly identical clinical and radiological features, including diffuse ground-glass opacities, peribronchovascular consolidation, and hypoxemia (20–22). Thus, during the diagnostic workflow, especially when SARS-CoV-2 RT-PCR testing yields negative results, a detailed evaluation of RP-ILD is critical, as it may represent the sole clinical manifestation at the onset of anti-MDA5^+^ DM. Given the symptomatic overlap between anti-MDA5^+^ DM and SARS-CoV-2 infection, misdiagnosis and treatment delays are potential risks. It is recommended that SARS-CoV-2 infection testing be routinely performed for patients with anti-MDA5 antibody positivity or other MSA, as well as those presenting with acute interstitial pneumonia, to facilitate timely and accurate diagnosis and management (17, 20, 23).

Multiple studies have indicated that cytokine levels are significantly higher in patients with anti-MDA5^+^ DM-related ILD or RP-ILD than in those without these complications. For example, the concentrations of cytokines such as interleukin-6 (IL-6), interleukin-8 (IL-8), interleukin-15 (IL-15), and monocyte chemoattractant protein-1 (MCP-1) are markedly elevated, mirroring the cytokine storm observed in severe SARS-CoV-2-induced pneumonia (24–27). Macrophage activation syndrome (MAS) represents a rare but recognized complication of anti-MDA5^+^ DM, with its clinical complexity potentially heightened by SARS-CoV-2 infection. These patients commonly exhibit elevated anti-MDA5 antibody titers, a hyperinflammatory state, and cytokine storm phenomena (28). In SARS-CoV-2-infected individuals, increased anti-MDA5 antibody levels may drive the production of IL-6 and IL-15, which are also prominently elevated in MAS. Therapies targeting interleukin-1 (IL-1) and IL-6 have demonstrated efficacy in treating MAS and have also improved symptoms in COVID-19 patients (29–32). Although the specific effects of SARS-CoV-2 infection on the progression of anti-MDA5^+^ DM or concomitant MAS remain unclear, evidence suggests that SARS-CoV-2 may exacerbate DM by triggering a predisposed inflammatory cascade in susceptible individuals.

A shared pathogenic feature of patients with anti-MDA5^+^ DM and SARS-CoV-2 infection is the activation of the type I interferon (IFN-I) signalling pathway. Considerable research has shown that the IFN-I system contributes critically to skin, muscle, and lung injury in anti-MDA5^+^ DM patients, with markedly elevated IFN-α levels detected in both serum and peripheral blood mononuclear cells (PBMCs). Elevated IFN-α concentrations may facilitate the development of severe RP-ILD (33–35). Additionally, studies have confirmed that RNA-containing immune complexes (ICs), formed by the MDA5 protein and anti-MDA5 antibodies, potently stimulate IFN-α production *in vitro* (36). The interplay between SARS-CoV-2 infection and the IFN-I signalling pathway is highly complex. Recent investigations have shown that MDA5-mediated recognition of SARS-CoV-2 specifically and robustly induces the production of both IFN-I and IFN-III. These interferons inhibit viral replication across diverse cell types; however, in primary lung epithelial cells, the IFN response is insufficient to suppress viral replication completely (37, 38). Significant delays or dysfunctions in the IFN system may lead to an exacerbated inflammatory response in patients (39–42).

#### 2.2.3 Anti-SARS-CoV-2 vaccines elicit the production of anti-MDA5 antibodies in healthy individuals

Some investigators have proposed that anti-SARS-CoV-2 mRNA vaccines may be associated with anti-MDA5 antibody production. The evidence suggests that these vaccines primarily induce an IFN-I response and can activate RNA sensors, including Toll-like receptor 7 (TLR7) and MDA5 (43). Multiple case reports have documented the emergence of anti-MDA5 antibodies in genetically predisposed healthy individuals following the administration of anti-SARS-CoV-2 mRNA vaccines (44–47). Additionally, a recent large-scale cohort study in Yorkshire, UK, revealed that SARS-CoV-2 vaccination coverage in the region reached 90% by 2021. Notably, this timeframe coincided with a significant increase in the incidence of anti-MDA5^+^ DM, suggesting a temporal association between vaccination and disease onset (6).

#### 2.2.4 The impact of SARS-CoV-2 infection on the prognosis of anti-MDA5^+^ DM

A critical clinical question is whether superimposed SARS-CoV-2 infection exacerbates anti-MDA5^+^ DM and leads to adverse outcomes. Emerging evidence reveals a complex and seemingly paradoxical picture. A large retrospective cohort study conducted during the Omicron-predominant period found that, compared to other IIM subtypes-particularly ASS, patients with anti-MDA5^+^ DM did not exhibit a higher risk of COVID-19-related hospitalization, severe disease, or mortality (48). This relatively favorable prognosis in this cohort may be attributed to two factors: first, a “survivor bias”, as the enrolled anti-MDA5^+^ DM patients had survived the initial high-risk phase of RP-ILD and exhibited stable pulmonary function; second, the potential protective effect of pre-exposure to Janus kinase (JAK) inhibitors, a commonly used regimen for anti-MDA5^+^ DM, which has been independently associated with a reduced risk of severe COVID-19 among hospitalized IIM patients (48).

However, this observation should not be interpreted as evidence of inherent resistance to SARS-CoV-2. On the contrary, substantial evidence indicates that SARS-CoV-2 infection can act as a potent trigger for catastrophic deterioration in anti-MDA5^+^ DM. Case reports have documented SARS-CoV-2 infection directly precipitating fatal RP-ILD in patients with anti-MDA5^+^ DM (17). Moreover, the presence of anti-MDA5 antibodies in otherwise healthy individuals with COVID-19 has been associated with more severe pulmonary inflammation and worse radiological outcomes (16), suggesting a potential pathogenic role of these autoantibodies in the context of viral infection. Given that both SARS-CoV-2 infection and anti-MDA5^+^ DM are characterized by hyperactivation of the IFN-I pathway and robust cytokine release, their co-occurrence may result in synergistic immune dysregulation, accelerating the progression of ILD and potentially triggering MAS (28).

In conclusion, the impact of SARS-CoV-2 infection on the prognosis of anti-MDA5^+^ DM is context-dependent. While patients with stabilized disease receiving immunosuppressive therapies-such as JAK inhibitors-may exhibit relative resilience to severe COVID-19, SARS-CoV-2 infection remains a significant threat for patients with new-onset, active, or uncontrolled anti-MDA5^+^ DM. In these individuals, the infection may trigger RP-ILD through amplification of shared immunopathological mechanisms.

### 2.3 Anti-MDA5^+^ DM and other viral infections

The contribution of other viral infections to the pathogenesis and progression of anti-MDA5^+^ DM remains a critical research focus. Through extensive research, it has been shown that numerous members of the herpesvirus family encode proteins capable of activating RNA sensors, with retinoic acid-inducible gene I (RIG-I) and MDA5 being prime examples (49, 50). Furthermore, during viral replication, HSV-1 generates RNA intermediates that can serve as nucleic acid ligands for RLRs, thereby activating the host’s antiviral immune response (51). Given these findings, the possible association between double-stranded DNA herpesviruses and anti-MDA5^+^ DM has been the subject of considerable scrutiny.

Human cytomegalovirus (HCMV), a beta-herpesvirus, is a significant cause of morbidity and mortality in immunocompromised individuals (52). Emerging evidence suggests an association between HCMV and anti-MDA5^+^ DM, although the underlying mechanisms require further elucidation. Studies indicate that HCMV reactivation is frequent in DM patients and may correlate with increased disease severity and mortality (53–55). A landmark study demonstrated a higher prevalence of HCMV infection in this patient group, alongside a significantly reduced one-year survival rate in those with HCMV DNAemia. Furthermore, lower counts of CD4^+^ T cells and CD19^+^ B cells were observed in anti-MDA5^+^ DM patients with CMV-IgM positivity (56). These findings suggest that HCMV may contribute to the pathogenesis and poor prognosis of anti-MDA5^+^ DM by impairing immune function.

Herpes simplex virus type 1 (HSV-1), an alpha-herpesvirus, primarily causes oral and perioral infections but exhibits enhanced invasiveness and recurrence in immunocompromised hosts (57). HSV-1 has been detected in bronchoalveolar lavage fluid (BALF) from patients with nonspecific interstitial pneumonia (NSIP) and idiopathic pulmonary fibrosis (IPF), indicating its potential involvement in idiopathic ILD (58). Additionally, case reports have described severe reactivation of HSV-1 infection in a DM patient receiving methotrexate therapy (59). IFN, a key cytokine family, plays a central role in inhibiting HSV-1 replication. Studies have demonstrated a significant upregulation of the “HSV-1 infection” pathway in DM and NSIP (60). The reactivation of HSV-1 initiates a cascade of antiviral responses, including elevated IFN levels (61). Dysregulation of these responses may lead to exacerbated inflammation, thereby facilitating disease progression. Although evidence regarding HSV-1 infection in patients with anti-MDA5^+^ DM-related ILD remains limited, its potential mechanistic relevance warrants further investigation.

### 2.4 Potential mechanism by which viral infection induces the production of anti-MDA5 antibodies

Previous studies have suggested that viral infections may trigger the production of anti-MDA5 antibodies, although the exact mechanism underlying this association remains unclear. Following viral exposure, potential pathways include MDA5 antigen overexpression, hyperactivation of the innate immune pathway, molecular mimicry, and persistent viral infection, which can lead to a breakdown of immune tolerance. Collectively, these mechanisms are hypothesized to drive the generation of anti-MDA5 antibodies.

#### 2.4.1 Overexpression of the MDA5 antigen and overactivation of the innate immune pathway

MDA5 mRNA overexpression has been observed in anti-MDA5^+^ DM PBMCs (33). Notably, MDA5 expression is significantly upregulated not only in lung tissue from anti-MDA5^+^ DM patients but also in samples from individuals with IPF (62, 63). Further research revealed that soluble MDA5 protein can be detected in the serum of patients with anti-MDA5^+^ DM. When human PBMCs are stimulated with polyinosinic-polycytidylic acid (poly(I:C)), MDA5 protein expression increases, and soluble MDA5 protein containing the helicase domain is rapidly released from the cytoplasm (64). Thus, viral infections may induce tissue injury (e.g., in the lungs and skin), leading to the release of MDA5-related proteins. These proteins are then captured by antigen-presenting cells (APCs) and presented to the immune system, where they activate autoreactive T and B cells, which ultimately results in the production of anti-MDA5 antibodies.

**Figure 1:**
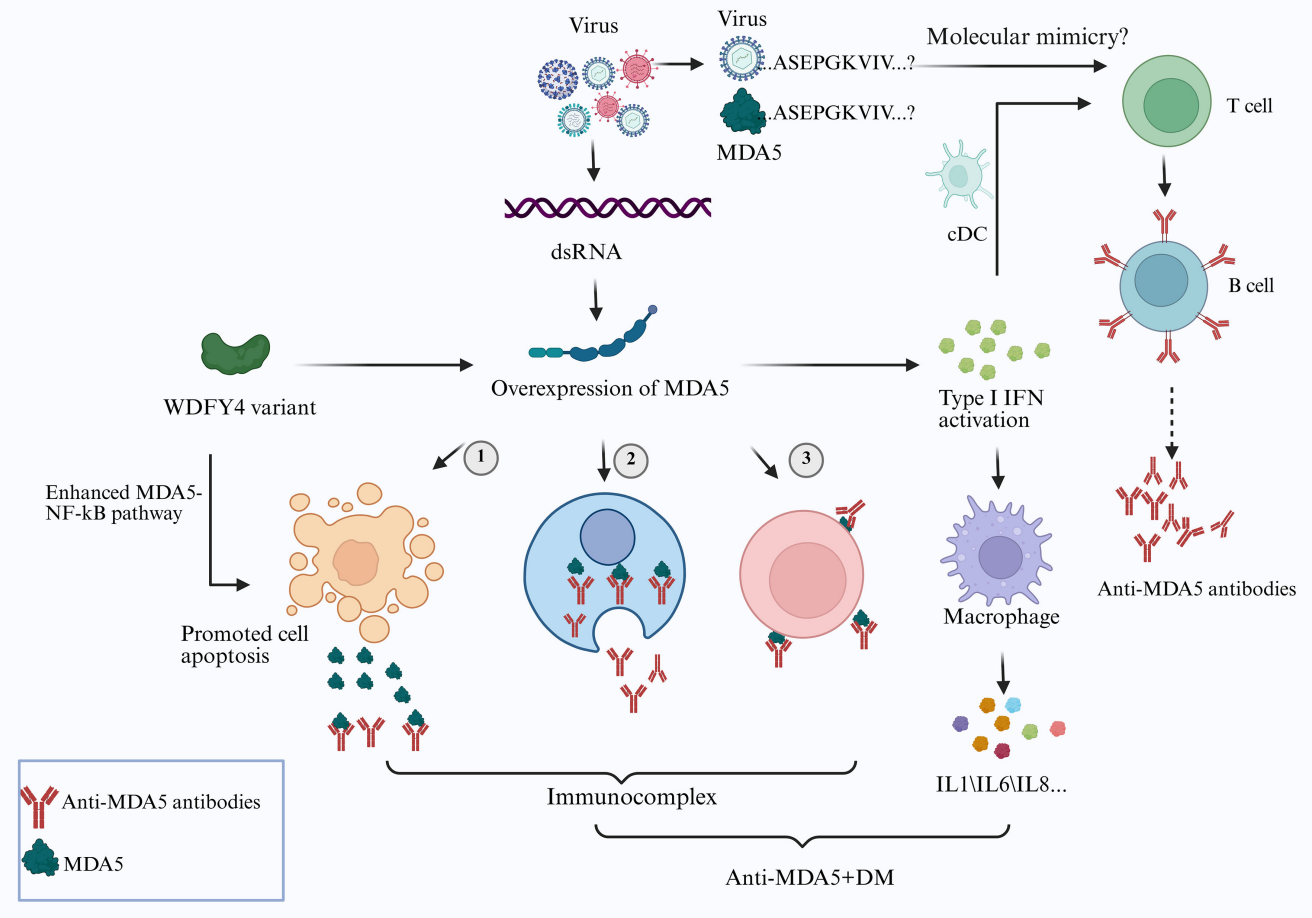
Mechanistic schematic of anti-MDA5 antibody production and viral infection-driven pathogenesis. Viral infection induces the overexpression of melanoma differentiation-related gene 5 (MDA5), triggering robust type I interferon (IFN-I) production, increasing the function of antigen-presenting cells (e.g., conventional dendritic cells (cDCs)) and enhances T-cell responses, ultimately promoting B-cell differentiation to produce anti-MDA5 antibody-secreting plasma cells. Certain viral antigens exhibit structural or sequence homology with MDA5, promoting immune system cross-reactivity and the generation of autoantibodies. Additionally, WDFY4 variants enhance MDA5-mediated NF-κB pathway activation, accelerating apoptosis in virus-infected cells and facilitating the extracellular release of MDA5. Anti-MDA5 antibodies bind to MDA5 released from apoptotic cells, intracellular MDA5, or cell surface-exposed MDA5 to form immune complexes. Concurrently, IFN-I stimulates macrophages to secrete proinflammatory cytokines (e.g., IL-1, IL-6, and IL-8). These immune complexes and inflammatory factors collectively contribute to tissue damage (Created with bioRender.com).

As previously described, MDA5, an innate immune recognition receptor, specifically recognizes viral double-stranded RNA (dsRNA), thereby activating the downstream RNA-MDA5 signalling pathway and substantially increasing IFN-α/β production (65, 66). Elevated IFN levels have also been observed in patients with anti-MDA5^+^ DM (33–35). Multiple potential mechanisms for anti-MDA5 antibody production have been proposed: individuals with specific genetic backgrounds (such as HLA-DRB1*04:01, *04:05, *12:02, *12:01 (67–70) and WDFY4 variants (71–73)) may exhibit constitutive activation of the RNA-MDA5 signalling pathway even in the absence of known viral infections, leading to robust induction of IFN-I. IFN-I further amplifies MDA5 expression through positive feedback loops. Apoptotic cells release MDA5, whereas specific specialized cells secrete MDA5 into the extracellular space. Concurrently, IFN-α enhances antigen presentation by APCs, such as classical dendritic cells (cDCs), and promotes T-cell and B-cell responses, ultimately triggering autoantibody generation against MDA5. (**Figure 1**).

A role for WDFY4 variants in the pathogenesis of anti-MDA5^+^ DM has been proposed, with associations observed between these variants and the production of anti-MDA5 antibodies. Kochi et al. conducted a large-scale genome-wide association study (GWAS) in Japan and identified a significant correlation between the WDFY4 locus (rs7919656) and clinically amyopathic dermatomyositis (CADM). Both full-length WDFY4 and its truncated isoform (tr-WDFY4) interact with pattern recognition receptors (PRRs), including MDA5 and TLR3, 4, and 9, to activate the nuclear factor kappa B (NF-κB) signalling pathway. Notably, tr-WDFY4 enhances MDA5-mediated NF-κB pathway activation, inducing apoptosis in virus-infected cells and subsequently releasing MDA5 into the extracellular space (72). (**Figure 1**) Theisen et al. later confirmed that WDFY4 is critical for antigen cross-presentation by mouse conventional type 1 dendritic cells (CD1c^+^) (73). Guo et al. demonstrated that variants of WDFY4 upregulate WDFY4 expression in the peripheral blood and lung tissue of Chinese patients with anti-MDA5^+^ DM-related RP-ILD (71). Collectively, these findings suggest that qualitative and/or quantitative alterations in WDFY4 may promote MDA5 release via virus-infected cell apoptosis and disrupt antigen cross-presentation pathways, potentially triggering aberrant autoimmune responses.

Recent studies have provided direct experimental evidence for the ‘two-hit’ pathogenesis model -in which poly(I:C)-mediated viral mimicry triggers overt disease in the context of pre-existing anti-MDA5 autoimmunity-using a novel murine model of ILD (74). In this model, initial immunization with MDA5 protein alone successfully induced anti-MDA5 antibody production and expansion of MDA5-reactive T cells but failed to elicit significant pulmonary pathology. Crucially, subsequent intranasal challenge with poly(I:C), a synthetic double-strand RNA analog that mimics viral infection, triggered prolonged respiratory inflammation and progressive fibrotic remodeling in MDA5-primed mice-key histopathological features that closely recapitulate human anti-MDA5^+^ DM-related ILD. Notably, in contrast to MDA5-immunized wild-type mice, MDA5-immunized IFN-α receptor (IFNAR)-null mice rarely developed ILD features following poly (I:C) administration. This study mechanistically integrates three critical pathogenic components: MDA5 overexpression, innate immune activation (interferon production), and the indispensable role of viral infection in transitioning from subclinical autoimmunity to clinical overt, progressive pulmonary disease.

#### 2.4.2 Molecular mimicry

Studies have shown that the pathogenic spike glycoprotein of SARS-CoV-2 shares linear sequence motifs with proteins in the human proteome (75, 76). Recently, three immunogenic linear epitopes identified in DM patients with anti-TIF1-γ antibodies were found to exhibit high similarity to sequences in the SARS-CoV-2 proteome (77). These findings suggest a potential molecular mimicry mechanism for the production of anti-MDA5 antibodies, whereby viral antigens display structural or sequence homology to MDA5, leading to immune system cross-reactivity and subsequent autoantibody generation. (**Figure 1**). While no direct sequence homology between the SARS-CoV-2 and human MDA5 proteins has been reported thus far, the role of molecular mimicry in anti-MDA5^+^ DM warrants further exploration.

#### 2.4.3 Persistent viral infection and disruption of immune tolerance

Certain viruses, including HSV-1 and HCMV, are capable of establishing latent infections. During latency, viral antigens are presented via major histocompatibility complex (MHC) molecules, leading to chronic stimulation of the immune system and potential disruption of immune tolerance (78, 79). Notably, HCMV DNA detection rates are significantly higher in anti-MDA5^+^ DM patients than in healthy controls, indicating that persistent viral infection may contribute to disease pathogenesis (56). An abnormal accumulation of viral RNA, potentially resulting from replication defects or delayed clearance, could continuously activate the MDA5-IFN pathway. This chronic activation may promote B-cell differentiation into plasma cells and drive the production of anti-MDA5 antibodies.

## 3 Anti-MDA5 antibody-positive dermatomyositis: anti-MDA5 antibody

### 3.1 Anti-MDA5 antibody and MDA5 antigen

In 1990, early reports from Japanese investigators described cases of ILD associated with CADM, even in patients receiving intensive immunosuppressive therapy; however, the overall prognosis remained poor (80). In a seminal study, Sato et al. utilized advanced immunoprecipitation and immunoblotting techniques in 2005 to identify a novel antibody reactive with a 140 kDa antigen in 50-70% of CADM patients (2). The CADM 140 antigen was subsequently confirmed to be identical to the previously characterized MDA5 in 2009 (81, 82).

**Figure 2:**
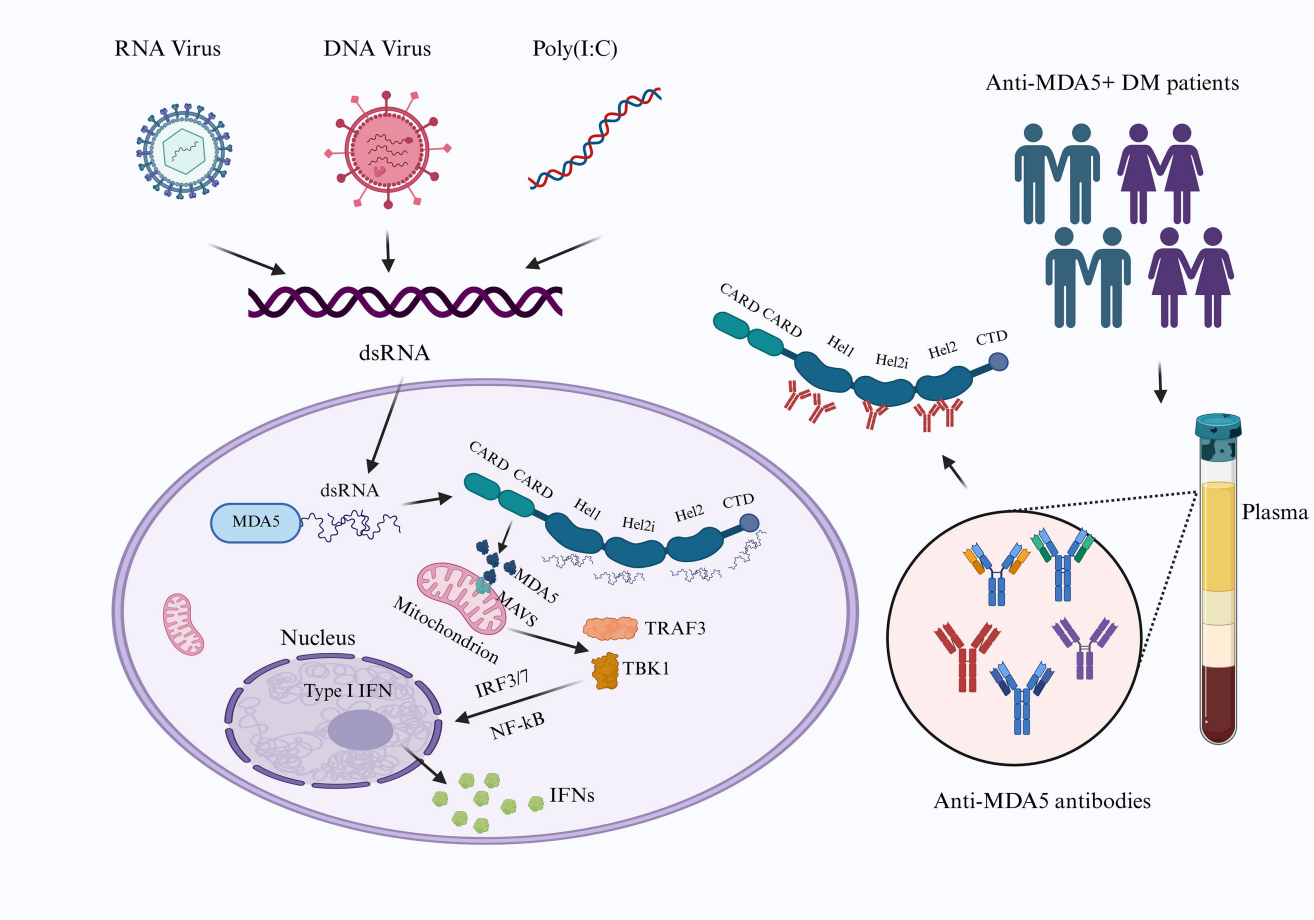
MDA5-MAVS signalling pathway and anti-MDA5 antibody epitopes. The C-terminal domain (CTD) and helicase domains of MDA5 recognize viral double-stranded RNA (dsRNA) or analogs such as poly(I:C), inducing conformational changes in MDA5 that expose its caspase activation and recruitment domain (CARD, triggering the activation of the mitochondrial antiviral signalling protein (MAVS), initiating a signalling cascade via TRAF3 and TBK1. The cascade culminates in the activation of IRF3, IRF7, and NF-κB, driving IFN-I production. In anti-MDA5^+^ DM patients, these autoantibodies localize primarily to the plasma and target the helicase domain of the MDA5 antigen (Created with bioRender.com).

Kang et al. (82) identified MDA5 as an IFN-inducible gene in human melanoma cells, demonstrating its double-stranded RNA-dependent ATPase activity (82). Subsequent research revealed that MDA5 is encoded by the interferon-induced helicase C domain one gene (IFIH1) and belongs to the retinoic acid-inducible gene 1-like receptor (RLR) family. As a cytoplasmic pattern recognition receptor, MDA5 specifically recognizes viral RNA for intracellular sensing, playing a critical role in the production of IFN-I and antiviral immune responses (83). More specifically, MDA5 primarily detects long double-stranded RNA (more than 300 bp), which commonly serves as a replication intermediate in RNA viruses. Notably, certain DNA viruses also generate such intermediates during their replication cycles (65, 84). For example, MDA5 can identify a broad range of RNA viruses, including picornaviruses, flaviviruses, and coronaviruses, as well as detect replication intermediates of herpesvirus family DNA viruses (84–87). (**Figure 2**).

The molecular architecture of MDA5 comprises two caspase activation and recruitment domains (CARDs), an RNA helicase domain featuring a DExD/H box motif, and a C-terminal domain (CTD). The RNA helicase domain is composed of three RecA-like subdomains: helicase 1 (HEL1), helicase 2 (HEL2), and the HEL2 insertion fragment (HEL2i) (83). Upon recognition of viral dsRNA by its RNA helicase domain and CTD, MDA5 engages with the adaptor molecule mitochondrial antiviral signalling protein (MAVS) through CARD-CARD interactions to propagate signals (66). This signalling cascade triggers the activation of the transcription factors interferon regulatory factor 3 and 7 (IRF3 and IRF7) as well as NF-κB via tumor necrosis factor (TNF) receptor-associated factor (TRAF) 3/6 and IκB kinase (IKK) family molecules (88–90). Phosphorylated IRF3, IRF7, and NF-κB then translocate to the nucleus to mediate the transcriptional regulation of antiviral genes, including IFN-I, leading to the induction of IFN-β and proinflammatory cytokine genes (83, 91, 92) (**Figure 2**).

The cellular localization and secretion of MDA5 under specific conditions have been a key focus of investigations. Berger et al. reported the predominant expression of MDA5 in the cytoplasm, secretory vesicles, and cell surface of neutrophils (93). Okamoto et al. recently demonstrated the presence of MDA5 in the serum of anti-MDA5^+^ DM patients, with soluble MDA5 protein containing helicase domains being released from the cytoplasm of PBMCs upon stimulation with the RNA analog poly(I:C) (64). These findings suggest that MDA5 may localize to the cytoplasm and the cellular surface or be secreted into the extracellular milieu.

### 3.2 Pathogenic mechanisms of anti-MDA5 antibodies

Serum anti-MDA5 antibody titers have been shown to be correlated with clinical phenotypes, disease relapse, and prognosis (9, 94, 95). However, the underlying pathogenic mechanisms of anti-MDA5 antibodies remain unclear. Several potential pathogenic mechanisms have been proposed. First, these antibodies play a direct pathogenic role. Previous studies have demonstrated their capacity to directly induce enhanced formation of neutrophil extracellular traps (NETs) (96). The association between NETs and DM-ILD has been confirmed in multiple investigations. In DM-ILD patients, reduced DNase I activity leads to incomplete degradation of excessive NETs, which may then stimulate plasmacytoid dendritic cells (pDCs) to release IFN-I and drive increased cytokine expression (97–102). Moreover, components within NETs, such as neutrophil granule proteins and extracellular matrix proteins, may exert a direct cytotoxic effect on endothelial cells (103–106).

Moreover, the interaction between anti-MDA5 antibodies and the MDA5 antigen is central to the immune pathogenesis of anti-MDA5^+^ DM. Three potential mechanisms have been proposed: 1) MDA5 antigens are released from apoptotic cells into the extracellular space by specific cell types, where they form immune complexes with anti-MDA5 antibodies, ultimately contributing to tissue injury (72). 2) Certain cells internalize anti-MDA5 antibodies through endocytosis, allowing them to bind to cytoplasmic antigens and trigger downstream signal transduction cascades. The evidence supports the ability of these antibodies to penetrate cells and induce damage (107–109). 3) Under specific conditions, MDA5 antigens may translocate to the cell membrane, where they interact with anti-MDA5 antibodies secreted by B cells, thereby activating signalling pathways such as IFN-I production. As previously noted, Berger et al. confirmed the cell surface expression of MDA5 on neutrophils (93) (**Figure 1**).

Beyond these antigen-dependent mechanisms, emerging functional studies have demonstrated that patient-derived monoclonal autoantibodies can induce proinflammatory cytokine production (e.g., interferon-gamma, IFN-γ) independently of MDA5 antigen recognition, potentially through Fc receptor engagement or modulation of innate immune cell function (110). This highlights the functional heterogeneity of autoantibodies and suggests that antigen-independent pathways may also contribute to disease pathogenesis, expanding our understanding of how autoantibodies drive inflammation in anti-MDA5^+^ DM.

Recent studies have shown that when wild-type mice and transgenic mice with lung-specific overexpression of full-length human MDA5 were administered anti-MDA5 polyclonal antibodies, wild-type mice did not develop lung injury comparable to that of their transgenic counterparts. These findings suggest a critical interaction between anti-MDA5 antibodies and the overexpressed MDA5 protein, which may act as a pathogenic driver in the progression of anti-MDA5^+^ DM-related ILD (63). The RNA immune complexes (ICs) formed by the MDA5 antigen and anti-MDA5 antibody engage with TLR7, and *in vitro* experiments have demonstrated that these ICs increase IFN-α production by plasmacytoid dendritic cells (36). Using multiple approaches, investigators have detected anti-MDA5 autoantibodies in the serum of patients with anti-MDA5^+^ DM, demonstrating that these antibodies predominantly bind to the helicase domain of MDA5 in a native conformation-dependent manner. This binding may disrupt the canonical antiviral function of MDA5 as an RNA sensor and impair downstream IFN-I pathway regulation (111, 112). Collectively, these findings clarify the pathogenic mechanism by which the anti-MDA5 antibody-MDA5 protein complex drives the progression of anti-MDA5^+^ DM-related ILD or RP-ILD.

### 3.3 Anti-MDA5 antibodies are related to disease activity

A significant association exists between anti-MDA5 antibodies and the clinical phenotypes of patients with these antibodies. Compared with patients without anti-MDA5 antibodies, those with positive anti-MDA5 antibodies commonly exhibit cutaneous rash, ulcers, calcinosis, mechanic’s hands, ILD, and arthralgia/arthritis, along with higher mortality rates. In contrast, patients without anti-MDA5 antibodies tend to experience more severe myositis (113). The severity of manifestations, particularly ILD and skin ulcers, is closely correlated with anti-MDA5 antibody titers (114). Studies indicate that elevated anti-MDA5 antibody levels may contribute to the development of concurrent RP-ILD (8). The concentration of anti-MDA5 antibodies is positively correlated with the level of Krebs Von den Lungen-6 (KL-6), a highly sensitive biomarker for assessing ILD severity (115, 116). Multiple reports have confirmed high anti-MDA5 antibody levels in juvenile dermatomyositis (JDM) patients with RP-ILD. A landmark study demonstrated that patients with anti-MDA5 antibody levels exceeding 500 U/ml develop deep ulcers and necrotic crusts, whereas those with levels below 500 U/ml present with isolated or superficial ulcers without symptoms of skin necrosis (114).

The titer of anti-MDA5 antibodies serves as a diagnostic tool for evaluating disease activity. The measurement of anti-MDA5 antibodies is a reliable biomarker for monitoring disease remission, relapse, and prognosis in DM patients (9, 94, 95). For example, enzyme-linked immunosorbent assay (ELISA)-based quantification has shown significant reductions in anti-MDA5 antibody titers during disease remission. Conversely, the reappearance or elevation of anti-MDA5 antibodies can predict disease relapse (94). Additionally, studies have reported that elevated serum anti-MDA5 antibody levels are associated with poor prognosis, particularly increased mortality rates due to the progression of RP-ILD (9, 95).

### 3.4 Isotypes and subclasses of anti-MDA5 antibodies

Anti-MDA5 antibody isotypes and immunoglobulin G (IgG) subclasses are likely critical contributors to DM pathogenesis. Analysis of antibody isotypes (IgG, IgA, and IgM) against MDA5 in patient serum revealed that anti-MDA5 IgG and IgA are the predominant subtypes, with minimal detection of anti-MDA5 IgM. Among the anti-MDA5 IgG subclasses in patient serum, IgG1 and IgG4 are the primary subclasses identified. Specifically, a positive correlation has been observed between IgG1 levels and serum ferritin levels, as well as between the incidence and mortality rates of acute interstitial pneumonia. Co-positivity for anti-MDA5 IgG1 and IgG4 may predict mortality in patients with DM-ILD (117). Additionally, a cohort study demonstrated an association between anti-MDA5 IgG1/IgG3 positivity and prognosis in DM-ILD patients, with high anti-MDA5 IgG1 levels confirmed as an independent risk factor for poor prognosis (118).

Previous studies have demonstrated that macrophages are the primary source of cytokines (e.g., interleukin-6 (IL-6), interleukin-10 (IL-10), and interleukin-18 (IL-18)), as well as ferritin, which are closely correlated with disease severity, particularly in ILD/RP-ILD patients (119, 120). Anti-MDA5 IgG1-mediated effects may occur via antibody-dependent cell-mediated cytotoxicity (ADCC), with macrophages acting as effector cells. Thus, the interaction between anti-MDA5 IgG1 and macrophages likely contributes significantly to the pathogenesis of DM/CADM-related ILD.

### 3.5 Anti-MDA5 antibody co-occurs with other autoantibodies

In anti-MDA5^+^ DM patients, autoreactive B cells produce not only pathogenic anti-MDA5 antibodies but also other autoantibodies, such as anti-RO-52 and anti-SFPQ (splicing factor proline/glutamine-rich) antibodies. The coexistence of anti-MDA5 antibodies with these additional autoantibodies and their potential association with poor prognosis, particularly an increased risk of ILD or RP-ILD, has become a critical focus of current clinical research.

Ro-52, also referred to as tripartite motif protein 21 (TRIM21), belongs to the TRIM protein family and has multiple functions. This protein is involved in critical intracellular processes, including immune responses and protein degradation, where it serves an indispensable function (121). In multiple studies of anti-MDA5^+^ DM patients, the co-occurrence of anti-RO52 antibodies has been shown to significantly increase the risk of ILD, particularly RP-ILD (121–127). The concomitant presence of both anti-MDA5 and anti-RO-52 antibodies is associated with markedly reduced patient survival rates, as detailed in **Table 1**. The mechanism by which the combination of anti-RO-52 and anti-MDA5 antibodies enhances patient mortality remains undefined.

**Table 1:** Comparative analysis of disease activity and mortality in anti-MDA5 antibody-positive dermatomyositis patients with and without anti-RO-52 antibodies.

Country	Study (Year)	Cohort size(n)	Anti-Ro52 antibody status, %(n)	ILD, %(n)	RP-ILD, %(n)	Mortality, %(n)	Ref
China	2021	83	Positive 74.7%(62/83)	–	54.8%(34/62)	40.1%(25/62)	(122)
			Negative 25.3%(21/83)	–	23.8%(5/21)	14.3%(5/21)	
China	2022	87	Positive 62.1%(54/87)	–	51.8%(28/54)	64.8%(35/54)	(123)
			Negative 37.9%(33/87)	–	21.2%(7/33)	36.4%(12/33)	
India	2022	21	Positive 38.1%(8/21)	62.5%(5/8)	25.0%(2/8)	75.0%(6/8)	(126)
			Negative 61.9%(13/21)	53.8%(7/13)	15.3%(2/13)	15.3%(2/13)	
China	2023	246	Positive 64.2%(158/246)	92.4%(146/158)	48.7%(77/158)	29.1%(46/158)	(124)
			Negative 35.8%(88/246)	85.2%(75/88)	12.5%(11/88)	15.9%(14/88)	

SFPQ, also termed PTB-related splicing factor (PSF), is a member of the Drosophila behavior/human splicing (DBHS) protein family. This protein executes diverse molecular functions in the cell nucleus, participates in regulating gene expression, and directly binds to viral RNA during innate immune responses to combat viral infections (128). A study reported that 53% of patient sera are concurrently positive for anti-MDA5 and anti-SFPQ antibodies (14). Moreover, anti-SFPQ antibody positivity has been observed in anti-MDA5^+^ DM patients during disease relapse (14, 129). Thus far, the underlying mechanisms connecting the production of anti-SFPQ and anti-MDA5 antibodies remain incompletely understood.

Importantly, the functional heterogeneity of autoantibodies extends beyond serological coexistence. Studies using patient-derived monoclonal autoantibodies suggest that the co-occurrence of different autoantibodies (e.g., anti-MDA5 with anti-Ro52 or anti-SFPQ) may exert synergistic or additive inflammatory effects through distinct mechanisms. For instance, antibodies capable of complement activation and Fc receptor engagement could collectively amplify tissue damage (130). Future research should investigate whether autoantibody combinations exhibit distinct functional profiles compared to isolated antibodies, potentially explaining the worse prognosis observed in patients with multiple autoantibody positivity.

## 4 Therapeutic targeting of the type I interferon pathway in anti-MDA5^+^ dermatomyositis

Substantial experimental evidence supports a central role for IFN-I pathway activation in the pathogenesis of anti-MDA5^+^ DM. Key clinical studies have consistently demonstrated markedly elevated IFN-I levels in patient sera and PBMCs compared to healthy individuals or other myositis subtypes (33–35, 131). Furthermore, IFN-stimulated gene (ISG) expression profiles are significantly upregulated in peripheral blood, skin, muscle, and most critically, pulmonary tissues of affected individuals (132, 133). This robust and sustained IFN-I signature resembles an antiviral response and is mechanistically linked to end-organ damage, particularly the progressive fibrotic changes observed in RP-ILD.

The therapeutic relevance of this pathway is underscored by recent clinical trials evaluating JAK inhibitors (e.g., Tofacitinib, Upadacitinib). As downstream blockers of the IFN signalling cascade, JAK inhibitors have shown significant clinical benefit in patients with refractory anti-MDA5^+^ DM, particularly in stabilizing or improving RP-ILD progression. The following table (**Table 2**) summarizes key clinical trials that form the evidence base for this therapeutic strategy.

**Table 2:** Summary of clinical studies on JAK inhibitors in anti-MDA5 antibody-positive dermatomyositis.

Study (Year)	Study design	Patient population (n)	JAK inhibitor used	Key findings/outcomes	Ref.
2018	Single-center, retrospective case series	Patients with refractory anti-MDA5^+^ DM-ILD (n=5)	Tofacitinib (10 mg/day)	The first study demonstrated the use of TOF against refractory anti-MDA5^+^ DM-ILD.	(143)
2019	Single-center, open-label, prospective study with historical controls	Patients with early-stage anti-MDA5^+^ DM-ILD (n=18)	Tofacitinib (5 mg twice daily)	18/18 (100%) survival at 6 months vs 78% in controls (P = 0.04). Significant improvements in ferritin, FVC, and DLCO.	(144)
2020	Single case report	Patient with recurrent anti-MDA5^+^ DM-ILD (n=1)	Tofacitinib (10 mg/day)	Effective as remission re-induction therapy after relapse. Improved skin ulcers and stabilized ILD.	(145)
2023	Prospective, single-arm, open-label trial	Patients with new-onset, untreated anti-MDA5^+^ DM (n=15)	Tofacitinib (5 mg twice daily)	71.4% (10/14) of patients responded to treatment at 6 months. Significant increase in total lymphocyte count (p=0.045) and CD8^+^ T cells	(146)
2023	Retrospective case series	Patients with refractory anti-MDA5^+^ JDM (n=9)	Tofacitinib (based on body weight)	55.5% (5/9) of patients showed alleviated or vanished ILD after a median follow-up of 14.5 months.	(147)
2024	Single case report	Patients with refractory anti-MDA5^+^ DM-RP-ILD (n=1)	Tofacitinib (10–20 mg/day)	Switching from tacrolimus to tofacitinib (initial 10 mg/day, later increased to 20 mg/day) led to disease remission.	(148)
2025	Single case report	Patients with refractory anti-MDA5^+^ DM-RP-ILD (n=1)	Tofacitinib (10 mg/day)	Addition of tofacitinib rescued a critically ill patient, but was associated with a pulmonary embolism.	(149)
2025	Multicentre, retrospective cohort study	Adults with newly diagnosed anti-MDA5^+^ DM-ILD (n=515; TOF: 290, CNI: 225)	Tofacitinib	Tofacitinib demonstrated superior 1-year survival compared to calcineurin inhibitors, reducing the risk of death or lung transplantation by 28% with a comparable safety profile.	(150)

TOF, tofacitinib; FVC, forced vital capacity; DLCO, diffusing capacity of the lungs for carbon monoxide; JDM, Juvenile Dermatomyositis; ILD, interstitial lung disease; RP-ILD, rapidly progressive ILD; CNI, calcineurin inhibitor.

Collectively, the convergence of experimental and clinical data supports a unifying model of disease pathogenesis and treatment. The consistent detection of an IFN-I signatures in patients provides a mechanistic rationale for therapeutic targeting, while the clinical validation of JAK inhibitors offers direct translational evidence of this approach’s efficacy. This synergy positions the IFN-I pathway as a critical driver of disease and a promising therapeutic target in anti-MDA5^+^ DM. The preliminary success of JAK inhibition underscores the interplay between antiviral immunity and autoimmune dysregulation in this disease, offering a life-saving intervention for a historically high-mortality patient population.

The management of anti-MDA5^+^ DM, particularly its life-threatening complication of RP-ILD, demands an aggressive, multimodal therapeutic strategy. While JAK inhibitors represent a mechanism-driven approach targeting the IFN-I pathway, high-dose glucocorticoids remain the cornerstone of initial therapy (3). Emerging evidence favors intensive upfront combination regimens over step-up strategies. A multicenter, prospective study demonstrated that triple therapy (high-dose glucocorticoids, intravenous cyclophosphamide, and calcineurin inhibitors) significantly improved 6- and 12-month survival compared to step-up regimens, albeit at the cost of higher HCMV reactivation rates (85% vs. 33%) (134). Retrospective analyses corroborate these findings, linking triple therapy to improved pulmonary function but also elevated HCMV risk (135).

For refractory cases, adjunctive therapies such as B-cell depletion (rituximab) and plasma exchange (PE) have shown promise in select cohorts (136–139). Notably, approximately 50% of anti-MDA5^+^ DM patients develop intercurrent infections, predominantly within 3 months of diagnosis (140–142). Prompt identification and treatment of these infections are critical for optimizing outcomes.

Therapeutic decisions should balance disease severity, progression rate, infection risk, and individual patient factors. Future head-to-head trials are essential to clarify the optimal positioning, sequencing, and risk-benefit profiles of JAK inhibitors and other agents within the therapeutic algorithm for anti-MDA5^+^ DM.

## 5 Conclusion and future perspectives

Anti-MDA5^+^ DM is a distinct subtype of DM, defined by the presence of anti-MDA5 antibodies and RP-ILD. The COVID-19 pandemic has been linked to an increased incidence of this condition, prompting significant global research interest. This observation suggests that viral infections may trigger the production of anti-MDA5 antibodies and disease onset, although direct experimental evidence remains limited.

The pathogenesis of anti-MDA5^+^ DM is proposed to involve a positive feedback loop initiated by viral infections, comprising four sequential stages: 1) The viral infection initiation phase: viruses, such as SARS-CoV-2 and herpesviruses, are recognized by the pattern recognition receptor MDA5 via their dsRNA, activating the MAVS-IRF3/7 pathway and driving the robust production of IFN-I. 2) The autoantibody generation and amplification phase: IFN-I enhances the function of antigen-presenting cells (e.g., cDCs) and activates autoreactive B cells in genetically susceptible individuals (e.g., those with HLA-DRB1*04:05), leading to the production of anti-MDA5 antibodies (predominantly IgG1/IgG4 subtypes). 3) Immune complex formation and inflammation phase: Anti-MDA5 antibodies bind to MDA5 released from apoptotic cells, forming RNA-containing ICs. These ICs further activate IFN-α through immune signalling molecules such as TLR7 and stimulate macrophages to release proinflammatory cytokines (e.g., IL-6 and IL-15). 4) End-organ damage phase: IC deposition in lung tissue induces ILD or RP-ILD through mechanisms such as NET formation and endothelial cell injury. This cycle mechanistically links three key clinical features: the seasonal disease onset pattern, antibody titer-prognosis correlation, and persistent IFN-I signatures in refractory RP-ILD.

The clinical management of patients with anti-MDA5^+^ DM-related ILD or RP-ILD presents significant challenges. Approximately 40% of RP-ILD patients have a poor response to conventional immunosuppressive therapies, highlighting the urgent need for targeted interventions. Current efforts are focused on modulating key pathogenic pathways, such as the use of anti-interferon alpha and beta receptor subunit 1 (anti-IFNAR1) antibodies to block IFN-α signalling and TLR7 inhibitors to disrupt immune complex-mediated inflammation.

Future investigations should prioritize three key areas: 1) Translational animal models: the development of preclinical models that recapitulate lung injury patterns in anti-MDA5^+^ DM, enabling mechanistic studies and therapeutic testing. Current models lack fidelity to human disease, particularly in replicating the IFN-driven autoantibody-immune complex cascade. 2) Multiomic biomarker discovery: Identification of predictive biomarkers for RP-ILD via integrated genomics, proteomics, and immunomics approaches. Candidate markers may include circulating immune complex profiles, NET-related peptides, or IFN-I signature gene expression patterns. 3) Combination targeted therapies: Exploration of combinatorial strategies, such as IC clearance agents paired with NET inhibitors. Preclinical data suggest that dual targeting of IC-mediated TLR7 activation and NET-induced tissue injury may synergize to mitigate lung fibrosis. These initiatives aim to bridge the gap between mechanistic insights and clinical application, ultimately improving outcomes for patients with this severe autoimmune phenotype.
